# Predicting potential miRNA-disease associations based on more reliable negative sample selection

**DOI:** 10.1186/s12859-022-04978-3

**Published:** 2022-10-17

**Authors:** Ruiyu Guo, Hailin Chen, Wengang Wang, Guangsheng Wu, Fangliang Lv

**Affiliations:** 1 School of Software, East China Jiaotong University, Nanchang, 330013 China; 2 School of Mathematics and Computer Science, Xinyu University, Xinyu, 338004 China

**Keywords:** miRNA-disease association predictions, Supervised learning, Negative sample selection

## Abstract

**Background:**

Increasing biomedical studies have shown that the dysfunction of miRNAs is closely related with many human diseases. Identifying disease-associated miRNAs would contribute to the understanding of pathological mechanisms of diseases. Supervised learning-based computational methods have continuously been developed for miRNA-disease association predictions. Negative samples of experimentally-validated uncorrelated miRNA-disease pairs are required for these approaches, while they are not available due to lack of biomedical research interest. Existing methods mainly choose negative samples from the unlabelled ones randomly. Therefore, the selection of more reliable negative samples is of great importance for these methods to achieve satisfactory prediction results.

**Results:**

In this study, we propose a computational method termed as KR-NSSM which integrates two semi-supervised algorithms to select more reliable negative samples for miRNA-disease association predictions. Our method uses a refined *K*-means algorithm for preliminary screening of likely negative and positive miRNA-disease samples. A *Rocchio* classification-based method is applied for further screening to receive more reliable negative and positive samples. We implement ablation tests in KR-NSSM and find that the combination of the two selection procedures would obtain more reliable negative samples for miRNA-disease association predictions. Comprehensive experiments based on fivefold cross-validations demonstrate improvements in prediction accuracy on six classic classifiers and five known miRNA-disease association prediction models when using negative samples chose by our method than by previous negative sample selection strategies. Moreover, 469 out of 1123 selected positive miRNA-disease associations by our method are confirmed by existing databases.

**Conclusions:**

Our experiments show that KR-NSSM can screen out more reliable negative samples from the unlabelled ones, which greatly improves the performance of supervised machine learning methods in miRNA-disease association predictions. We expect that KR-NSSM would be a useful tool in negative sample selection in biomedical research.

**Supplementary Information:**

The online version contains supplementary material available at 10.1186/s12859-022-04978-3.

## Background

As one category of endogenous non-coding RNAs with about 20–24 nucleotides in length, miRNAs have been widely discovered in plants, viruses and human beings [1]. miRNAs function as regulators of gene expression by binding to the 3ʹ-untranslated region (UTR) of their target mRNAs, which would cause translational repression or transcript degradation [2]. Existing studies have revealed that miRNAs are implicated in many crucial processes [3, 4], such as cell proliferation, apoptosis, development, differentiation and metabolism. Therefore, the dysregulation of miRNAs would result in a large number of diseases [5]. Currently, miRNAs have been recognized as important biomarkers for disease diagnosis, and detection of disease-related miRNAs can contribute to the pathological studies of diseases.

As traditional biological experiments are time consuming and costly, computational methods to determine potential associations between miRNAs and diseases are emerging as efficient complementary tools. These methods are mainly based on the assumption that miRNAs with similar functions tend to be associated with similar diseases [6, 7]. For example, Chen et al. [8] analysed the effects of similarity measurements on miRNA-disease association prediction and presented a semi-supervised inference method NetCBI to prioritize associations between miRNAs and human diseases by combining OMIM phenotype similarity information and miRNA functional similarity information. Han et al. [9] proposed a novel method DismiPred to predict disease-related miRNA candidates by incorporating functional similarity and association information. Xuan et al. [10] developed a computational model MIDP by random walk on miRNA-disease bilayer network established based on similarity between nodes to predict disease-related miRNAs. Chen et al. [11] proposed a prediction model WBSMDA to combine within- and between-scores for potential miRNA-disease association inference. Chen et al. [12] developed a computational method HAMDA to uncover novel miRNA-disease associations by integrating network structure, node attribution and information propagation on bipartite miRNA-disease network. You et al. [13] proposed a prediction model PBMDA to infer potential miRNA-disease associations by adopting a depth-first search algorithm on miRNA-disease heterogeneous graph. Chen et al. [14] presented an inductive matrix completion model IMCMDA to complete missing miRNA-disease associations based on known miRNA-disease associations, integrated miRNA similarities and integrated disease similarities. Chen et al. [15] proposed a novel computational model BNPMDA for miRNA-disease association predictions based on bipartite network projection [16]. Xuan et al. [17] developed a method DMAPred which applied non-negative matrix factorization for potential miRNA-disease association inference. DMAPred projected miRNAs and diseases into low-dimensional spaces to yield feature representations. The likelihood that a miRNA was associated with a disease was calculated according to these projections. Chen et al. [18] proposed a recommendation-based computational framework MDVSI to predict miRNA-disease associations by incorporating miRNA topological similarity and functional similarity. Zhang et al. [19] developed a computational model MSFSP to predict disease-related miRNAs by similarity fusion and space projection. Wang et al. [20] developed an unbalanced random walk algorithm MGDF on genome-wide similarity networks to predict miRNA–disease associations. These similarity-based approaches have achieved encouraging miRNA-disease association prediction performance, and there still exists room for improvement.

Meanwhile, inspired by the successful application of machine learning methods in the fields of web searches, content filtering and e-commerce, many researchers have applied machine learning techniques to infer miRNA-disease associations. For example, Chen et al. [21] formulated the miRNA-disease association prediction as a classification problem and developed a decision tree-based method for association predictions. Feature vectors from existing associations including similarity measurement were used to train a regression tree under a gradient boosting framework for determining whether a miRNA-disease association existed or not. Chen et al. [22] proposed a random forest-based model to infer miRNA-disease associations, in which feature vectors to represent miRNA-disease samples were defined by integrated similarities, and their dimensions were further reduced for building an effective classifier. Zhao et al. [23] developed an adaptive boosting approach ABMDA for predicting potential associations between diseases and miRNAs. ABMDA improved learning accuracy by integrating weak classifiers constructed on decision trees. Peng et al. [24] proposed a learning framework MDA-CNN for miRNA-disease association identification. An auto-encoder was applied in their model to extract essential features and a convolutional neural network was used for prediction. Ji et al. [25] presented a network embedding-based method to predict miRNA-disease associations, in which the embedding representations of miRNA and disease were learned from a heterogeneous information network and the Random Forest (RF) classifier was used for predicting potential miRNA-disease associations. Liu et al. [26] developed a computational framework SMALF to infer possible miRNA-disease associations. SMALF utilized a stacked autoencoder to learn latent features. XGBoost was used to make predictions from the unlabelled miRNA-disease associations. Tang et al. [27] presented a graph convolutional network-based method MMGCN with multi-view multichannel attention to predict potential miRNA–disease associations. Liu et al. [28] proposed a computational method DFELMDA to predict miRNA-disease associations, in which two deep autoencoders were applied for low-dimensional feature representations and prediction scores of unlabelled miRNA-disease associations were received by deep random forest. Wang et al. [29] proposed a graph attention networks-based framework MKGAT and used dual Laplacian regularized least squares to predict potential miRNA-disease associations. With the recent advances in machine learning especially in deep learning, these methods have received more and more accurate results in miRNA-disease association predictions.

It is known that both positive and negative samples are needed for supervised machine learning methods to predict reliable miRNA-disease associations. However, the required negative samples are not available due to lack of research interest in life sciences. Previous studies used two strategies to address this problem. The first one is randomly selecting negative samples from the unlabelled associations [22, 26, 30]. The other one is dividing the unlabelled miRNA-disease samples into *K* parts using *K*-means algorithm, and randomly selecting negative samples from the *K* clusters [23, 31]. As positive samples exist in the whole unlabelled ones, the two selection strategies would bring noise and result in less reliable prediction performance.

In this study, we propose a novel mothed named KR-NSSM to select more reliable negative samples for miRNA-disease association inference. Specifically, KR-NSSM first combines similarity measurements from miRNAs and diseases to generate feature vectors for miRNA-disease pairs. It then applies SS-Kmeans [32] to obtain likely negative and positive samples from the unlabelled ones. *Rocchio* classification [33] is finally used to receive more reliable negative and positive samples for inference. Comprehensive experiments based on fivefold cross-validations show using negative samples received by our method KR-NSSM could significantly improve prediction accuracy compared with using these by existing negative sample selection strategies. Moreover, we obtain 1123 reliable positive samples by using KR-NSSM, among which 469 have been confirmed by existing databases.

## Results

### Evaluation metric

The benchmark datasets (see “Methods”) contain 5430 experimentally confirmed miRNA-disease associations, which are considered as positive samples in this study. We select negative samples from the unlabelled ones using not only our method KR-NSSM, but also existing methods, such as random selection or *K*-means. We test the effects of negative samples selected by different strategies on final predictions. We apply fivefold cross-validations to systematically analyse prediction performance, in which the samples are randomly divided into five equal parts. In each validation, one part is used as the test set and the other four parts as the training set. We prioritize the inferred miRNA-disease associations according to the final prediction results. True positive rate (TPR) and false positive rate (FPR) are calculated by varying the thresholds. We further calculate AUC, AUPR, Precision, Recall, F1-score and Accuracy as evaluation metric for performance assessment and comparison.

### Ablation test in KR-NSSM

In our method KR-NSSM, we combine SS-Kmeans and *Rocchio* classification for negative sample selection. To test whether this combination strategy helps infer miRNA-disease associations, we design three categories of ablation experiments. The first one is only using SS-Kmeans for screening. The second one is only using *Rocchio* classification for screening. The third one is integrating the two strategies for screening. We use logistic regression (LR) as a benchmark classifier and conduct fivefold cross-validations to test their prediction performance. The experiments are based on a balanced data set of positive and negative samples. The results are shown in Table 1. We can discover from Table 1 that using negative samples from KR-NSSM gets the best prediction performance, which indicates the negative samples received by KR-NSSM are the most reliable.

**Table 1:** The ablation experimental results based on fivefold cross-validations

model	AUC	AUPR	Precision	Recall	F1-score	Accuracy
SS-Kmeans	0.9712	0.9792	0.9531	0.8682	0.9081	0.9133
*Rocchio* classification	0.9652	0.9712	0.9398	0.8433	0.8886	0.8946
KR-NSSM	**0.9763**	**0.9811**	**0.9630**	**0.8751**	**0.9168**	**0.9208**

The bold value indicates the highest one in each column

### Performance evaluation on classic classifiers

In order to further evaluate the performance of our method KR-NSSM, we use six different classification algorithms for miRNA-disease association predictions. The six classifiers are: lightGBM, support vector machine (SVM), Random Forest (RF), logistic regression (LR), XGBoost and Multilayer perceptron (MLP). LightGBM is a computational framework implemented with gradient lifting decision trees (GBDT). We set the number of decision trees in lightGBM as 1000, the maximum number of leaf nodes as 100, the learning rate as 0.05, and the rest parameters as default values. SVM is a classical binary classification model, which has achieved good results in many classification problems. We use RBF kernel, and the remaining parameter values are set to be default in SVM. In random forest, we set the number of decision trees as 50, and the rest parameters as default values. In XGBoost, we set the number of trees to be 1000, the learning rate to be 0.1, and the remaining parameters as default values. For MLP, we set two hidden layers, each layer is 30 and 20 neurons respectively, and update the weights by using quasi-Newton method.

**Fig. 1:**
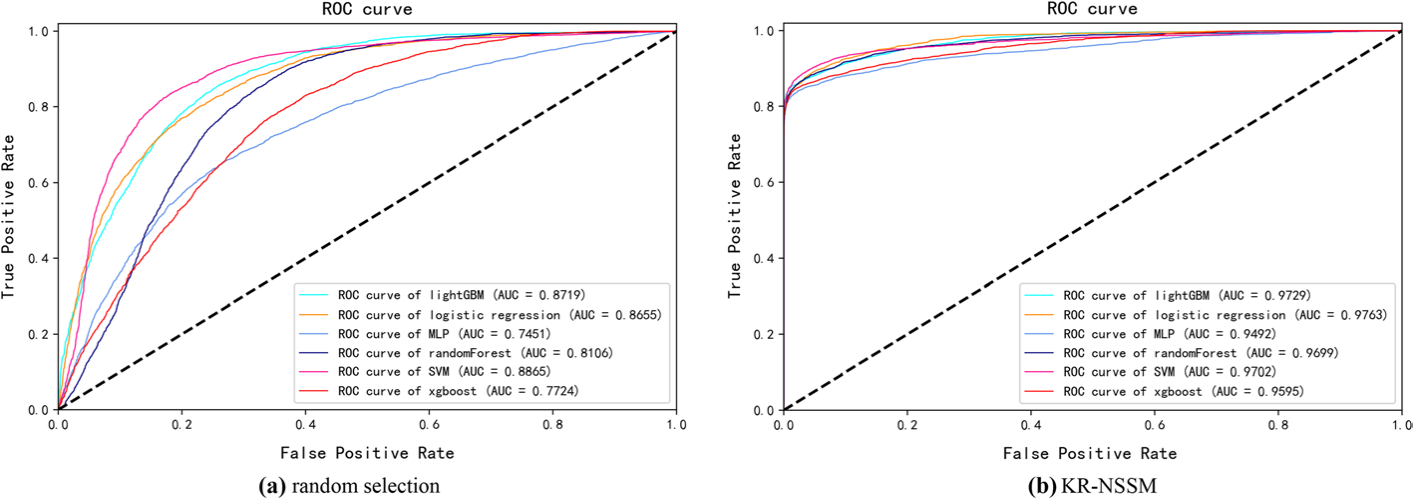
ROC curves of different classifiers based on fivefold cross-validations and different strategies of negative sample selection

**Fig. 2:**
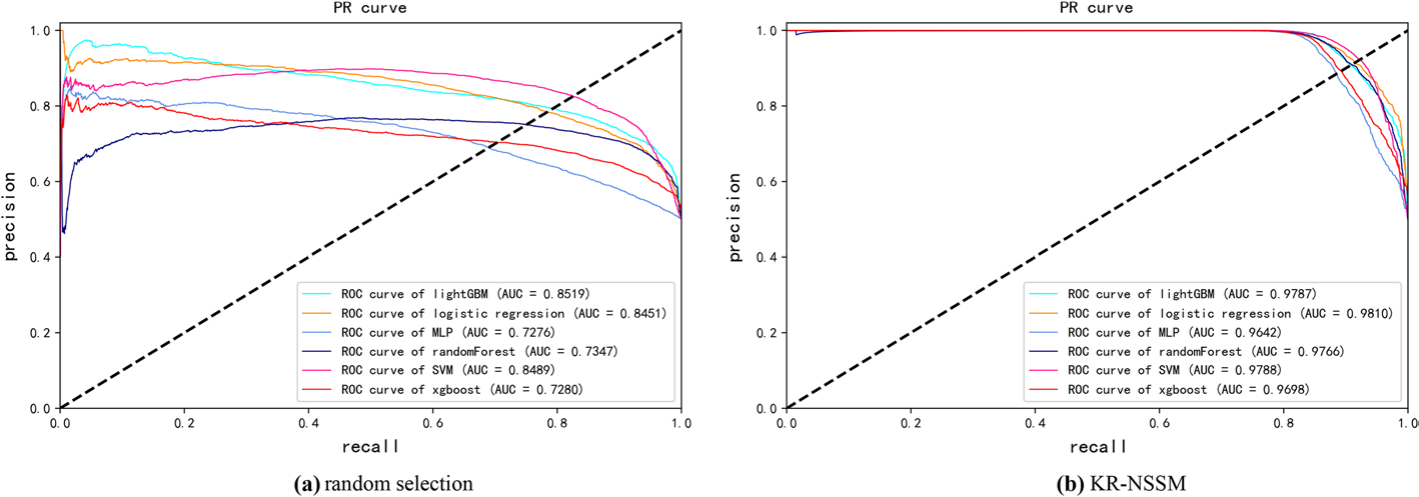
PR curves of different classifiers based on fivefold cross-validations and different strategies of negative sample selection

Since 5430 experimentally verified miRNA-disease associations are taken as positive samples in our study (see “Methods”), we use KR-NSSM to select 5430 negative samples to generate a balance data set. In the control group, we randomly choose 5430 negative samples from the unknown associations. We conduct fivefold cross-validations for association predictions and plot ROC and PR curves in Figs. 1 and 2, respectively. Table 2 lists the prediction performance. We can find from Table 2 that better performance is received when using negative samples by our method KR-NSSM, which indicates that the negative samples selected by KR-NSSM are more reliable.

**Table 2:** Performance comparison based on six classical classifiers and fivefold cross-validations

classifier	AUC	AUPR	Precision	Recall	F1-score	Accuracy
*KR-NSSM*						
lightGBM	0.9723	0.9787	0.9678	0.8681	0.9150	0.9196
SVM	0.9701	0.9788	0.9701	0.8799	0.9225	0.9263
RF	0.9699	0.9766	0.9731	0.8608	0.9131	0.9185
LR	0.9763	0.9810	0.9630	0.8751	0.9168	0.9208
XGBoost	0.9595	0.9698	0.9655	0.8554	0.9069	0.9125
MLP	0.9492	0.9642	0.9537	0.8527	0.9001	0.9056
*Random selection*						
lightGBM	0.8719	0.8519	0.8099	0.6853	0.7406	0.7629
SVM	0.8865	0.8489	0.8376	0.8015	0.8189	0.8230
RF	0.8106	0.7347	0.7438	0.4573	0.5599	0.6543
LR	0.8655	0.8451	0.8240	0.7004	0.7570	0.7754
XGBoost	0.7724	0.7280	0.7363	0.4042	0.5187	0.6315
MLP	0.7451	0.7276	0.7575	0.4860	0.5892	0.6669

### Performance evaluation on existing miRNA-disease association prediction models

We choose five existing supervised methods (RFMDA [22], IRFMDA [30], ABMDA [23], GBDT-LR [31] and SMALF [26]), which were developed for miRNA-disease association predictions, for performance evaluation. Note RFMDA, IRFMDA and SMALF randomly select negative samples from the unlabelled associations, while ABMDA and GBDT-LR select negative samples by performing random sampling based on *K*-means clustering on the unlabelled associations. We replace these negative sample selection strategies with KR-NSSM, and evaluate the prediction performance based on fivefold cross-validation experiments. Performance evaluation results are summarised in Table 3, which suggests using negative samples obtained by KR-NSSM can significantly improve prediction performance. It further demonstrates the reliability of negative sample selection by KR-NSSM.

**Table 3:** Performance comparison of existing prediction methods based on fivefold cross-validations

method	AUC	AUPR	Precision	Recall	F1-score	Accuracy
*KR-NSSM*						
RFMDA	0.9414	0.9606	0.9818	0.8424	0.9064	0.9134
IRFMDA	0.9671	0.9739	0.9591	0.8582	0.9054	0.9109
ABMDA	0.9732	0.9789	0.9971	0.8427	0.9129	0.9201
GBDT-LR	0.9633	0.9730	0.9625	0.8654	0.9111	0.9158
SMALF	0.9913	0.9931	0.9749	0.9507	0.9626	0.9648
*Original selection*						
RFMDA	0.7388	0.7034	0.6253	0.9548	0.7453	0.6912
IRFMDA	0.9267	0.9222	0.8447	0.8598	0.8521	0.8567
ABMDA	0.8841	0.8807	0.8152	0.7827	0.7908	0.8027
GBDT-LR	0.9274	0.9014	0.8315	0.8273	0.8302	0.8304
SMALF	0.9503	0.9472	0.8808	0.8931	0.8868	0.8860

### Identification of positive miRNA-disease associations

Besides negative sample selection, KR-NSSM can produce positive miRNA-disease associations (see “Methods”). We eventually obtain a reliable positive set which contains 1123 potential miRNA-disease associations after implementing KR-NSSM on the benchmark datasets. We choose two established databases HMDD V3.2 [34] and dbDEMC [35], which store miRNA-disease association entries received by text-mining from literature, for validation. We discover that 469 out of the 1123 associations are supported by the databases. Note these unconfirmed associations may exist in reality as our investigation of miRNAs’ roles in diseases is not complete. We provide the 1123 positive associations as an additional file (see Additional file 1) for further studies.

## Conclusions

For supervised machine learning methods to miRNA-disease association predictions, a core challenge is that experimentally-supported uncorrelated miRNA-disease pairs used as negative samples are not available. In this study, we propose a negative sample screening model KR-NSSM to solve the problem. Our method consists of two steps: a refined *K*-means for preliminary screening and a *Rocchio* classification-based procedure for further screening. Compared with the original *K*-means and *Rocchio* algorithms, we take the experimentally-confirmed miRNA-disease association pairs in HMDD V2.0 as positive samples for more accurate classification. The ablation test in KR-NSSM shows that integrating the two procedures would increase prediction accuracy.

Experimental results from six classic classifiers and five well-known prediction models based on fivefold cross validations prove that using the negative samples obtained by KR-NSSM can significantly improve the accuracy of miRNA-disease association predictions. It is because we integrate two semi-supervised algorithms in KR-NSSM, so that more reliable negative samples can be selected. Meanwhile, KR-NSSM can also screen a certain number of reliable positive samples based on the same principle. Some of the selected positive samples are verified by existing databases. The experiments show the effectiveness of our method. Since more association predictions, such as drug-target [36], drug-disease [37], and lncRNA-disease [38], exist in bioinformatics fields, and negative samples are not available in these situations. Reliable negative samples are also needed to be selected in supervised methods for the association predictions. We believe that KR-NSSM can be widely applied in these fields for negative sample selection.

## Methods

### Benchmark dataset

The benchmark dataset used in our study is downloaded from reference [26], in which known miRNA-disease associations are obtained from HMDD V2.0 [39]. These miRNA-disease associations are considered as positive samples. miRNA functional similarity scores computed in reference [40] are taken as miRNA-miRNA similarities. Disease-disease similarities are calculated according to their semantic values based on the MeSH database (http://www.ncbi.nlm.nih.gov/). We finally receive 5430 miRNA-disease associations including 495 miRNAs and 383 diseases.

### Method overview

#### Construction of feature vectors

We construct the feature vectors to represent miRNA-disease associations as follows: first, we obtain a 383-dimensional vector consisting of 383 disease similarity scores to represent each disease, and a 495-dimensional vector consisting of 495 miRNA similarity scores to represent each miRNA. Then, we represent each sample by an 878-dimensional feature vector consisting of the 383 disease similarity scores and 495 miRNA similarity scores as Eq. (1):

1 $$F_{miRNA - disease} = \left( {f_{1} ,f_{2} ,...,f_{495} ,f_{496} ,...,f_{878} } \right)$$

where (f1, f2, ⋯, f495) represents the 495 miRNA similarity scores, and (f496, ⋯, f878) denotes the 383 disease similarity scores. In this study, we regard the experimentally validated miRNA-disease associations as positive samples, the unknown miRNA-disease associations as unlabelled samples. Correspondingly, *P* and *U* are used to represent the positive sample set and unlabelled sample set.

#### KR-NSSM

**Fig. 3:**
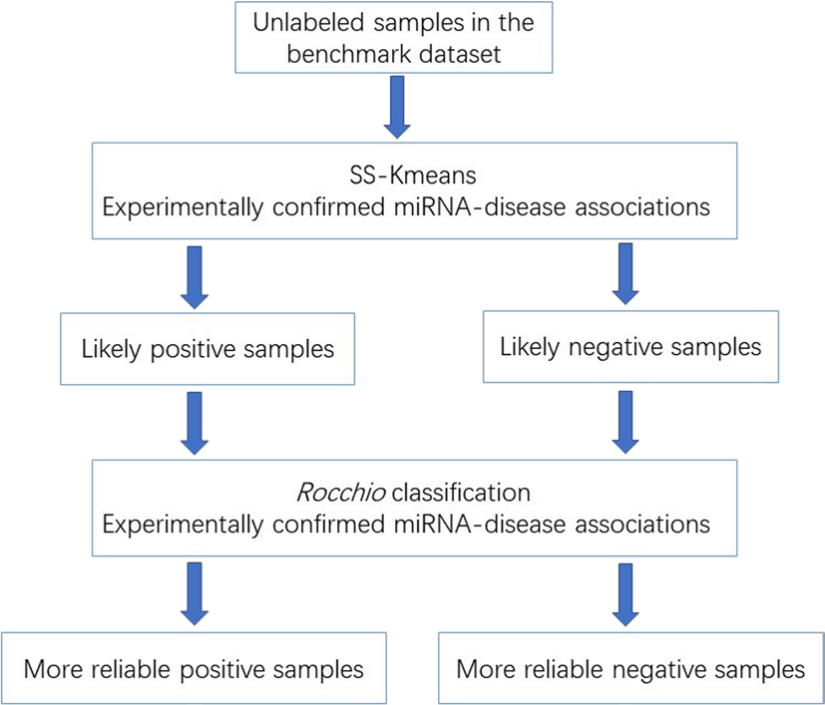
The workflow of our method KR-NSSM

Inspired by previous research [32, 33, 41], we propose a negative sample screening model KR-NSSM. The workflow of KR-NSSM is briefly shown in Fig. 3. We integrate two algorithms, i.e., SS-Kmeans and *Rocchio* classification, to construct the core framework of KR-NSSM. SS-Kmeans are applied to conduct preliminary screening on unlabelled samples, and then *Rocchio* classification are used to conduct further screening on the results of SS-Kmeans.

#### SS-Kmeans

In the first part of KR-NSSM, we use an improved *K*-means algorithm, SS-Kmeans [32], for screening. Different from the traditional unsupervised *K*-means algorithm, SS-Kmeans uses the information of both labelled and unlabelled samples. We first generate the centroid of positive sample set *P* and unlabelled sample set *U*, respectively. The centroid of positive sample $c_{1}$ is generated by all the feature vectors of *P*, and $c_{1}$ is calculated by Eq. (2)

2 $$c_{1} = \frac{{\sum\limits_{i = 0}^{m} {p_{i} } }}{m}$$

where $m$ is the number of positive samples, and $p_{i}$ represents the *i*th positive sample. Similarly, the sample set *U* are used to generate $c_{2}$, which is the centroid of reliable negative samples and is calculated as follows:

3 $$c_{2} = \frac{{\sum\limits_{j = 0}^{n} {u_{i} } }}{n}$$

where $u_{i}$ represents the unlabelled samples and $n$ is the number of unlabelled samples. We then compare the cosine similarity between each unlabelled sample $u_{i}$ and c_*k*_ as follows:

4 $$x_{i} = \arg \max_{k} \cos \mathrm{in}\, e(u_{i} ,c_{k} )$$

where *k* (= 1 or 2) represents $c_{1}$, or $c_{2}$, respectively. According to the value of cosine similarity, the unlabelled samples can be classified into likely positive sample set1(LP1) and likely negative sample set1(LN1).

In the third step, LP1 and LN1 are used to obtain new centroids where we denote them as $l_{1}$ and $l_{2}$, respectively. The new centroids are calculated according to Eq. (1) and Eq. (2). We use $l_{1}$ and $l_{2}$ for further classification. We apply the Euclidean distance to measure the similarity as follows:

5 $$x_{i} = \arg \min_{k} ||u_{i} - l_{k} ||^{2}$$

We repeat the steps until the latest centroids are stable. Eventually, we receive the likely positive sample set (LP1) and likely negative sample set (LN1) in SS-Kmeans.

#### Rocchio classification

In the second part of KR-NSSM, we use *Rocchio* classification [33] to further screen the preliminary results of SS-Kmeans. The core purpose of *Rocchio* classification is to generate two prototype vectors that represent positive sample set and negative sample set. More specifically, *Rocchio* classification can be subdivided into rocchio1 and rocchio2.

In the first step of *Rocchio* classification, *P* are regarded as positive sample set and we choose to use the experimentally confirmed miRNA-disease associations as *P*. *U* are regarded as negative sample set and we choose to use the LN1 (the likely negative sample obtained from SS-Kmeans) as *U*. The prototype vectors $\vec{c}^{ + }$ and $\vec{c}^{ - }$ are calculated by Eq. (6) and (7), respectively.

6 $$\vec{c}^{ + } = \alpha \frac{1}{|P|}\sum\limits_{{\mathop{d}\limits^{\rightharpoonup} \in P}} {\frac{{\vec{d}}}{{||\vec{d}||}}} - \beta \frac{1}{|U|}\sum\limits_{{\vec{d} \in U}} {\frac{{\vec{d}}}{{||\vec{d}||}}}$$

7 $$\vec{c}^{ - } = \alpha \frac{1}{|U|}\sum\limits_{{\vec{d} \in U}} {\frac{{\vec{d}}}{{||\vec{d}||}}} - \beta \frac{1}{{{|}P{|}}}\sum\limits_{{\vec{d} \in P}} {\frac{{\vec{d}}}{{||\vec{d}||}}}$$

where |*P*| and |*U*| is the number of samples in their correspond set. $||\vec{d}||$ is the binary norm of $\vec{d}$. $\alpha$ and $\beta$ adjust the relative influence of positive samples and negative samples and we set them to be 16 and 4, respectively.

Then, the samples in LN1 are classified according to their cosine similarity to prototype vectors. If the similarity between positive prototype vector and an unlabelled sample is less than that between negative prototype vector, the unlabelled sample will be classified as a reliable negative sample. Otherwise, a reliable positive sample. Eventually, we can form the reliable negative sample set2 LN2.

However, rocchio1 may still occur classification errors [33]. In order to solve the problem, we propose to use rocchio2. In rocchio2, the *K*-means algorithm are used to divide LN2 into multiple subsets, $i.e. \, N_{1} ,N_{2} ,N_{3} ,...,N_{k}$. For each subset, *P* will combine with them to form a pair of data set. The prototype vector is calculated by Eq. (8) and Eq. (9).

8 $$\vec{n}_{j} = \alpha \frac{1}{{|N_{j} |}}\sum\limits_{{\vec{d} \in N_{j} }} {\frac{{\vec{d}}}{{||\vec{d}||}}} - \beta \frac{1}{|P|}\sum\limits_{{\vec{d} \in P}} {\frac{{\vec{d}}}{{||\vec{d}||}}}$$

9 $$\vec{p}_{j} = \alpha \frac{1}{|P|}\sum\limits_{{\vec{d} \in P}} {\frac{{\vec{d}}}{{||\vec{d}||}}} - \beta \frac{1}{{|N_{j} |}}\sum\limits_{{\vec{d} \in N_{j} }} {\frac{{\vec{d}}}{{||\vec{d}||}}}$$

where $\vec{n}_{j}$ and $\vec{p}_{j}$ represent the *j*th pair of prototype vector. In this study, we use *K*-means to divide LN2 into 3 subsets. For each sample in LN2, we calculate the cosine similarity between it and each pair of prototype vectors. If the similarity between the sample and the negative prototype vector $\vec{n}_{j}$ is greater than that with the positive prototype vector $\vec{p}_{j}$, we consider it as a reliable negative sample.

## Supplementary Information

**Additional file 1.** The 1123 more reliable miRNA-disease associations selected by KR-NSSM.

## Data Availability

The datasets and source codes used in this study are available from the corresponding author on reasonable request.

## Abbreviations

UTR: Untranslated region
TPR: True positive rate
FPR: False positive rate
LR: Logistic regression
SVM: Support vector machine
RF: Random forest
MLP: Multilayer perceptron
GBDT: Gradient lifting decision trees
